# Exogenous lipoid pneumonia due to medical aspiration of paraffin oil: a case report and literature review

**DOI:** 10.3389/fmed.2025.1596160

**Published:** 2025-07-04

**Authors:** Mingshuang Li, Conglin Ren, Cangsong Chen

**Affiliations:** ^1^Taizhou Hospital, Shanghai University of Traditional Chinese Medicine, Taizhou, China; ^2^Taizhou Hospital of Traditional Chinese Medicine, Taizhou, China

**Keywords:** paraffin oil, respiratory aspiration, intubation, gastrointestinal, exogenous lipoid pneumonia

## Abstract

This case report describes a case of exogenous lipoid pneumonia (ELP) due to medical aspiration of paraffin oil. An 87-year-old male was hospitalized with bedridden, dysphagic dysphagia. Two days after being given nasal paraffin oil, the patient developed high fever and respiratory distress. Blood gas analysis showed a PaCO_2_ of 33 mmHg in the room air. CT scan of the chest showed multiple ground glass opacity with solid lesions. The patient then underwent bronchoscopy, and large quantities of oily turbid fluid was found in the bronchoalveolar lavage fluid (BALF). Further cytological analysis of the BALF showed 35% phagocytes, 60% neutrophils, and 5% lymphocytes. The patient was diagnosed with ELP based on a history of paraffin oil exposure, CT imaging of the chest, and cytological examination. Despite our aggressive anti-inflammatory and anti-infective treatment, the patient eventually passed away due to advanced age and multiple complications. Aspiration of oily substances is the most important risk factor for ELP. For people at high risk of misadministration, a suitable naso-intestinal tube is more appropriate for feeding and medication.

## Background

Lipoid pneumonia, characterized by the presence of lipid-laden macrophages or foam cells in the alveolar walls and interstitial tissues, can be divided into exogenous and endogenous lipoid pneumonia (1). Endogenous lipoid pneumonia, also known as cholesterol pneumonia, commonly occurs in chronic inflammatory diseases of the lungs. Localized inflammation leads to cell membrane disruption and intracellular lipid extravasation, which attracts macrophage aggregates that in turn affect lipid metabolism (2). Exogenous lipoid pneumonia (ELP) results from aspiration of lipid-rich substances, such as polyethylene glycol, paraffin oil, and diesel fuel, into the terminal fine bronchioles and alveoli (3). Lipids are phagocytosed by macrophages to form lipid-rich vacuolated cells, leading to sustained inflammatory response (4). Cryptogenic Organizing Pneumonia (COP) may be confused with ELP because of its wandering solid lesions, but COP has no history of lipid exposure and no lipid droplets in bronchoalveolar lavage fluid (BALF). Alveolar protein deposition resembles the “paving stone sign” on imaging, but the BALF is milky white. Therefore, it can be differentiated. Due to the lack of specificity in clinical symptoms and laboratory tests, many cases require chest imaging combined with a history of lipid aspiration to confirm the diagnosis. According to the literature, ELP is mostly induced by occupational exposure, repeated use of nasal drops, and autonomous use of drugs. While, medically induced aspiration is uncommon. Here, we report a case of medically induced lipoid pneumonia.

## Case presentation

**Figure 1:**
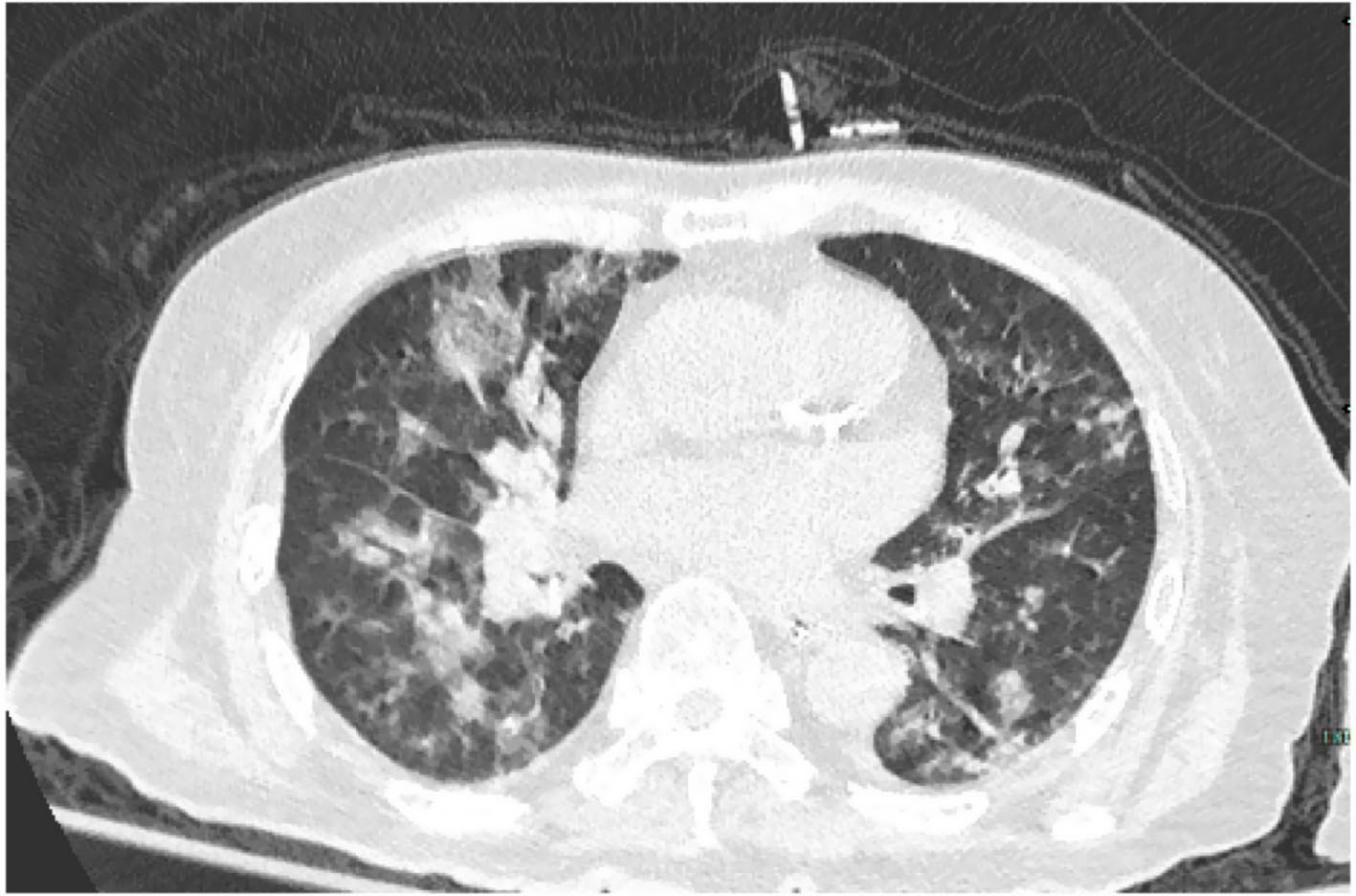
The chest CT scan showed patchy ground-glass lesions in the lungs, mainly along the bronchi.

**Figure 2:**
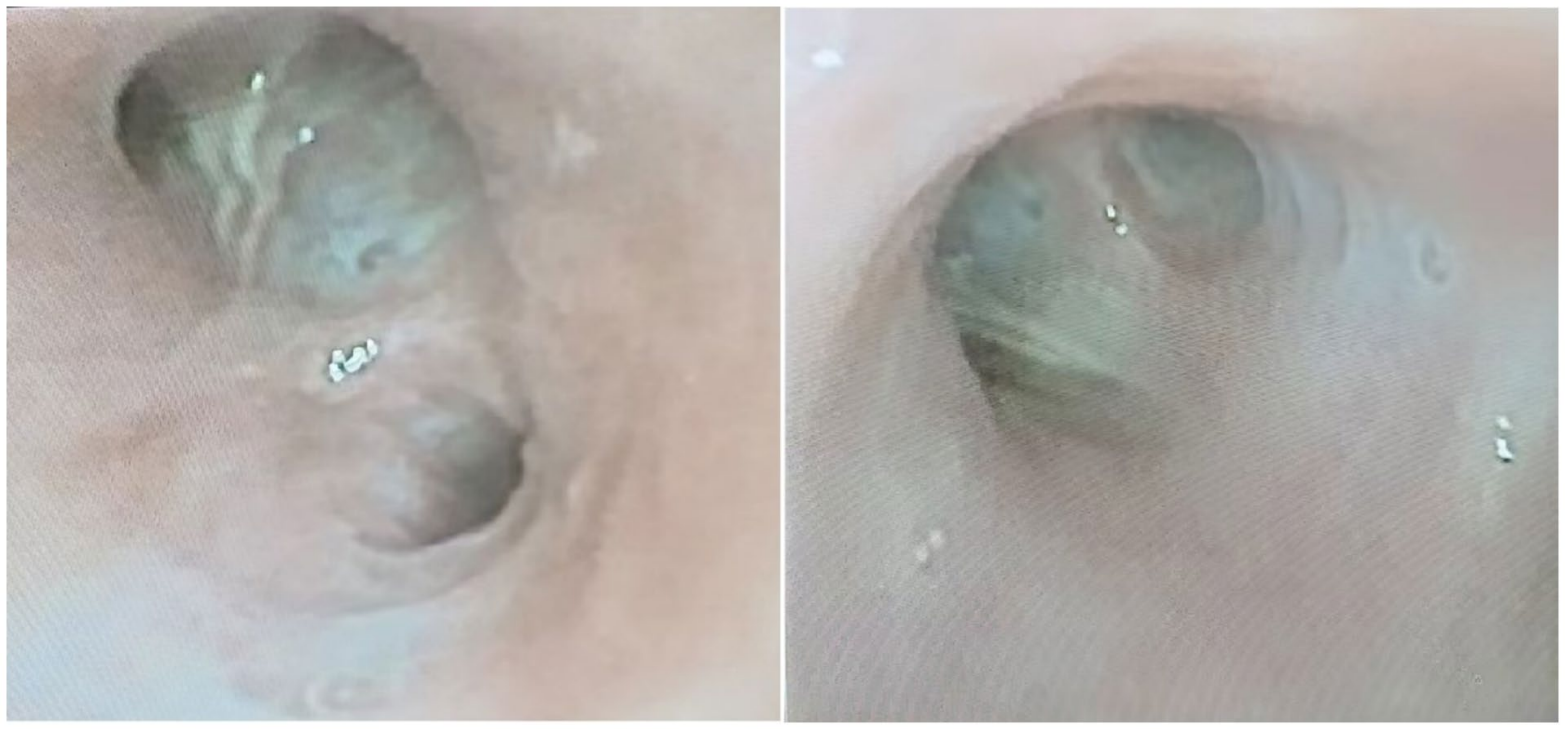
Bedside bronchoscopy showed large amounts of lipoidal mucus secretions distributed in the tracheal lumen.

**Figure 3:**
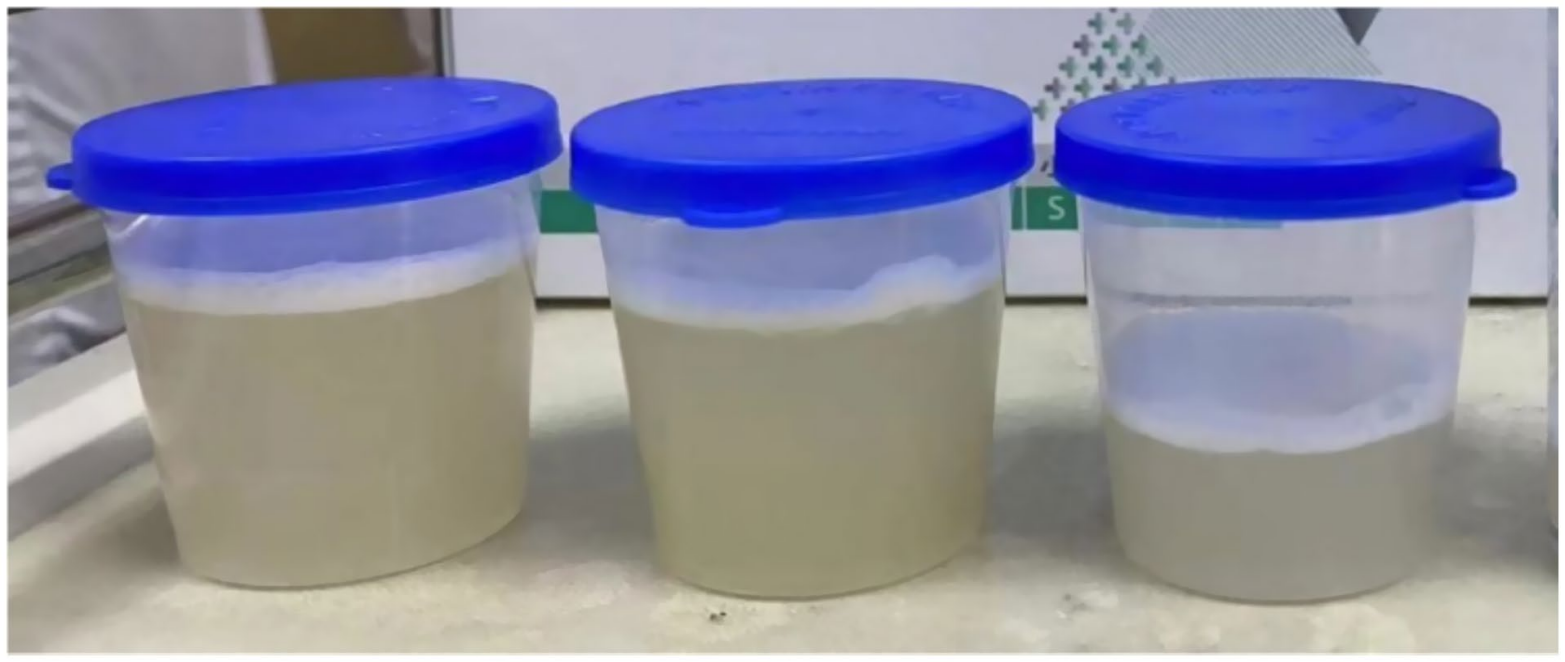
Large quantities of oily turbid fluid were found in the bronchoalveolar lavage fluid.

An 87- year-old male was admitted to the rehabilitation department of our hospital. He had a history of an old hip fracture and had been bedridden for more than 2 years. At the same time, the patient had a comorbid history of hypertension, Alzheimer’s disease, chronic bronchitis with emphysema, and chronic heart failure. Routine rehabilitation, including motor function exercises and swallowing training, was performed after admission to the hospital. On the 15th day of treatment, the condition worsened with abdominal pain and difficulty in defecation. Abdominal X-ray showed abundant contents in the intestinal cavity and scattered air-filled shadows. Therefore, a nasogastric tube was inserted through which 50 mL of paraffin oil was administered daily for laxative purposes. The patient passed a small amount of feces, but the nausea and vomiting did not subside. Two days later, the patient started to develop a fever with a maximum temperature of 38.9°C, accompanied by cough, expectoration and shortness of breath. Auxiliary examinations showed a high-sensitivity C-reactive protein of 127.79 mg/L, a white blood cell count of 9.5 × 10^9^/L, and a calcitoninogen of 0.239 ng/mL. Blood gas analysis showed a PaCO_2_ of 33 mmHg in the room air. Sputum culture showed the presence of *Pseudomonas aeruginosa* infection. However, after 2 days of treatment with ceftriaxone (2.0 g qd IV) prescribed by his physician, the clinical symptoms remained unrelieved. Surprisingly, a CT scan of the chest showed multiple ground glass opacity with solid lesions that were significantly worse than at the time of hospitalization. The CT measurements ranged between −120 and −50 Hounsfield Units (HU), with no change on enhanced examination. Both lungs were involved and there was an asymmetric distribution in both lower lungs, with small amounts of bilateral pleural effusion, consistent with acute ELP (Figure 1). After requesting a consultation with the Department of Pulmonary and Critical Care Medicine, the patient underwent bronchoscopy. As shown in Figures 2, 3, large quantities of oily turbid fluid were found in the BALF. Cytological analysis of the BALF showed 35% phagocytes, 60% neutrophils, and 5% lymphocytes. To exclude the possibility of fungal infection, we performed fungal culture and identification, galactomannan detection test, and fungal D-glucan detection test on BALF, all of which were negative. We also recommended pathological examination of BALF; regrettably, the patient’s family refused further testing. In summary, based on the patient’s medical history, chest CT imaging and cytological findings, we consider this to be a case of ELP. For treatment, we changed the nasogastric tube and treatment regimen, which included: methylprednisolone 40 mg bid IV, cefoperazone sulbactam 3.0 g q12h IV combined with levofloxacin 0.5 g IV. Despite aggressive treatment, the patient unfortunately passed away after the 12th day of the change in treatment regimen due to advanced age, worsening respiratory symptoms, and complications from an acute myocardial infarction.

## Discussion and conclusions

Swallowing dysfunction is known to be a high-risk factor for aspiration, which was further exacerbated in this case by the patient’s persistent nausea and vomiting. In clinical practice, all patients with nasogastric tubes should be assessed for the risk of aspiration. Tube placement should be carefully considered if the patient has any of the following conditions, including inability to protect the airway, mechanical ventilation, age >70 years, diminished consciousness, poor oral care, inadequate nurse-to-patient ratio, prolonged bed rest, neurologic deficits, and gastroesophageal reflux (5). In terms of nursing care, doctors should closely monitor the patient’s status and promptly address conditions such as nausea, vomiting, respiratory distress or fever (6). Therefore, a detailed history, such as the exposure to lipids, should be taken during the clinical consultation. The CT examination of ELP shows various manifestations, which can be a mass similar to lung cancer with unclear boundary; it can also be distributed bilaterally, mainly in the posterior segment of the right upper lobe, the right middle lobe and the two lower lobes, while pleural effusions are often described in acute ELP (7). Low-density solid lesions or nodules (CT values ranging from −150 to −30 HU) are characteristic of lipoid pneumonia (8). However, superimposed peripheral inflammation can lead to more prominent areas of fat hypodensity, resulting in solid or nodular shadows that are not readily observable in some patients, making diagnostic imaging more difficult (9). As with other diseases, pathological examination is the gold standard for the diagnosis of ELP. Currently, CT-guided percutaneous puncture lung biopsy is an effective test. The pathological manifestations of ELP include widening of the alveoli, the appearance of lipid vacuoles, and foam cells in the lumen of the fine bronchioles and alveolar septa, accompanied by lymphocytic infiltration and possibly fibrosis (10).

**Figure 4:**
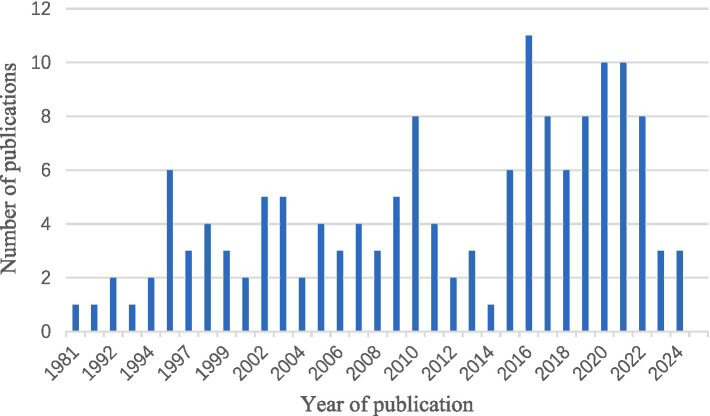
Trends in the growth of publications on lipoid pneumonia.

**Figure 5:**
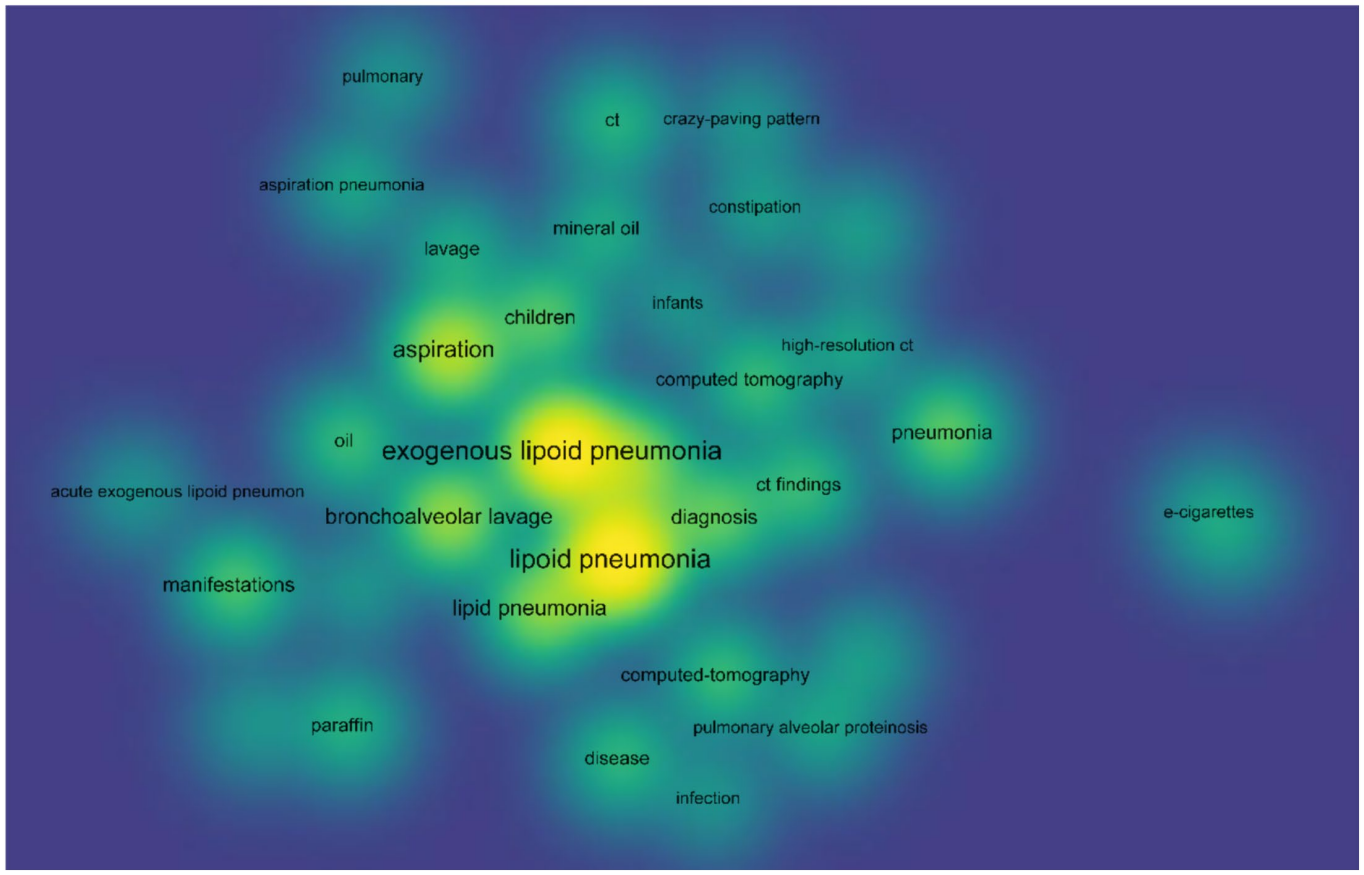
The density map of co-occurring keywords by VOS viewer.

In order to get a deeper understanding of the etiology of lipoid pneumonia, we conducted a retrospective study of literature. The keywords “lipoid pneumonia” or “exogenous lipoid pneumonia” or “endogenous lipoid pneumonia” were used to search for papers indexed in the Web of Science database between 1981 and 2024. After removing commentaries, book chapters and meeting papers, a total of 147 literatures were obtained. As can be seen in Figure 4, the number of articles on lipoid pneumonia has risen each year, with 11 papers published in 2017, indicating that clinicians are becoming more sensitive to lipoid pneumonia diagnosis. The top three countries with the highest number of publications were the United States of America, France and Italy, contributing 65 articles and 44% of the total (Table 1). CiteSpace analysis of the keywords revealed that “exogenous lipoid pneumonia” accounted for the largest proportion of cases, mostly due to aspiration of oily substances. It has also been noted that children are susceptible to this disease (Figure 5). We re-screened the above 147 articles, using “exogenous lipoid pneumonia” and “case report” as keywords, and obtained a total of 58 articles. After removing irrelevant and incomplete clinical data, we found 39 cases (Table 2).

**Table 1:** The top 10 productive countries with publications.

Rank	Countries	Article count	Percentage (*n* = 147)
1	USA	35	23.8%
2	FRANCE	16	10.9%
3	ITALY	14	9.5%
4	JAPAN	12	8.2%
5	SOUTH KOREA	10	6.8%
6	CHINA	9	6.1%
7	SPAIN	9	6.1%
8	GERMANY	7	4.8%
9	INDIA	7	4.8%
10	TURKEY	6	4.1%

**Table 2:** Literature review of cases of exogenous lipoid pneumonia.

case	country	References	Year	Age/sex	Risk factors	Aspiration	Symptoms	Complication	Treatment	Outcome
1	USA	Simmons et al. (15)	2007	72/female	Unknown	Mineral oil	Hemoptysis, cough, and dyspnea	Unknown	Unknown	Improved
2	Serbia	Tegeltija et al. (16)	2019	47/male	Smoke	Machine oil	Multifocal subpleural tumors	Unknown	Unknown	Partial improvement
3	Japan	Kuroyama et al. (11)	2015	38/female	NO	Sesame oil	Short of breath	NO	No Treatment	Improved
4	Japan	Kuroyama et al. (11)	2015	66/male	Smoke	Sesame oil	Dry cough	NO	Methylprednisolone And Bronchoalveolar Lavage	Much improved
5	France	Boutros et al. (12)	2019	83/female	Gastroesophageal reflux disease	Piascledine capsules (avocado/soybean unsaponifiables)	Dry nocturnal cough	Unknown	No Treatment	Improved
6	USA	McDonald et al. (13)	1994	5/Unkonw	Severe neurodevelop mental disease	Unknown	Respiratory failure	Unknown	Unknown	Died
7	India	Dikshit et al. (14)	2024	36/female	NO	Mustard (organic) oil	1-month history of low-grade fever, general malaise, and cough with scant expectoration	NO	Repeat Therapeutic Multisegmental Bronchoscopic Lavage	Much improved
8	Japan	Yamanaka et al. (17)	2025	46/male	NO	Kerosene	Nausea and dyspnea, accompanied by a cough	NO	Methylprednisolone	Much improved
9	France	Jouannic et al. (18)	1996	56/female	Chronic constipation	Liquid paraffin	Cough, whitish or purulent expectorations, exertional dyspnoea	*Mycobacterium fortuitum* and *Aspergillus fumigatus* infections	Antituberculosis Medication Regimen, Ciprofloxacin And Minocycline And Amphotericin B	Partial improvement
10	Japan	Gotanda et al. (19)	2013	91/female	Alzheimer’s disease	Kerosene	Coughing and hypoxia	Unknown	Antibiotherapy	Improved
11	Czech Republic	Doubkova et al. (20)	2013	38/female	NO	Baby body oil drops	NO	NO	No Treatment	Partial improvement
12	Turkey	Kara et al. (21)	2020	25/female	Cerebral palsy	Oil-based laxative	Cough, dyspnea and loss of appetite	Unknown	Antibiotherapy	Unknown
13	Italy	Vigo et al. (22)	2024	80/male	NO	Oil-based laxative	Exertional dyspnea and a dry cough	NO	Unknown	Unknown
14	China	Wang et al. (23)	2023	38/male	NO	Liquid paraffin	Fever, cough, sputum, chest tightness, and shortness of breath	*Mycobacterium abscessus*	Systemic Glucocorticoids And Anti-*M. abscessus* Treatment	Much improved
15	USA	Jeelani et al. (24)	2020	66/female	NO	Mineral oil	Dyspnea	NO	Antibiotics Therapy	Much improved
16	China	Shimizu et al. (25)	2020	25/female	NO	Vegetable oil	Fever, cough and short of breath	NO	Steroid Treatment	Improved
17	France	Chardin et al. (26)	2017	54/male	HIV	Snorting oily drugs	NO	NO	Lobectomy	Much improved
18	Poland	Siebert et al. (27)	2024	83/female	NO	Paraffin oil	Dyspnea, productive cough	NO	No Treatment	Improved
19	USA	Cabri et al. (28)	2017	57/female	NO	A mentholated petroleum-based topical ointment	Dry cough	NO	No Treatment	Improved
20	USA	Cabri et al. (28)	2017	66/male	NO	Mineral oil	Dyspnea, cough, and wheezing	NO	No Treatment	Improved
21	USA	Bandla et al. (29)	1999	6/male	Developmental delay	Mineral oil	NO	NO	Antibiotics Therapy	Improved
22	USA	Cohen et al. (30)	2003	55/female	Psoriasis	Petrolatum	Fever and dyspena	NO	Antibiotics And Steroid Therapy	Improved
23	South Korea	Gil et al. (31)	2019	65/female	NO	Squalene spray	Angina	nontuberculous mycobacterium	Ntm Medication	Improved
24	Japan	Yamada et al. (32)	2022	89/female	NO	Sesame oil	Sputum and cough	NO	Unknown	Improved
25	Turkey	Yigit et al. (33)	2010	19/male	NO	Paraffin oil	Fever, dyspnea, cough, and shortness of breath	Unknown	Unknown	Improved
26	Turkey	Yigit et al. (33)	2010	29/male	NO	Paraffin oil	Cough, fever, chest pain, and hemoptysis	Unknown	Antibiotics And Steroid Therapy	Improved
27	Italy	Terzano et al. (34)	2003	33/female	Multiple sclerosis	Paraffin oil	Exertional dyspnea, cough, and mild fever	NO	No	Partial improvement
28	India	Aurangabadkar et al. (35)	2024	55/male	NO	Diesel	Severe dyspnea, cough with mucoid expectoration with occasional blood-tinged sputum, fever, and right sided pleuritic chest pain	NO	Antibiotics And Corticosteroid	Partial improvement
29	Germany	Hübel et al. (36)	1998	54/male	NO	Oil substances	Cough, chest pain, and weight loss of 10 kg	NO	Right Upper Lobectomy	Much improved
30	France	Tahon et al. (37)	2002	72/male	Smoke	Vegetable oil	NO	NO	Right Lobectomy	Much improved
31	USA	Harris et al. (1)	2011	54/male	Gastroesophageal reflux disease	Unknown	Mild cough	NO	Right Lobectomy	Much improved
32	Spain	Haro et al. (38)	1998	71/male	Smoke	Paraffin oil	Massive haemoptysis	NO	Steroid Therapy	Much improved
33	USA	Kanaji et al. (39)	2008	76/male	Gastroesophageal reflux disease and smoke	Squalene spray	NO	*Mycobacterium tuberculosis*	No Treatment	Improved
34	Italy	Dell' Omo et al. (40)	2010	23/male	No	Iso-alkane mixture	Fever, dyspnoea, dry cough, intense right chest pain and occipital headache	NO	Antibiotics Therapy	Much improved
35	Italy	Ghezzi et al. (41)	2019	16/male	No	Petrochemical derivative	Chest tightness, cough, and fever	NO	Antibiotics And Steroid Therapy	Much improved
36	USA	Reddy et al. (42)	2021	56/male	No	Gasoline	Pleuritic chest pain and four episodes of hemoptysis hours	lung abscess	Thoracotomy	Partial improvement
37	China	Chen et al. (43)	2019	57/male	Smoke	Gasoline	Chest pain and shortness of breath	pulmonary fibrosis	Antibiotics, Steroid Therapies And Bronchoalveolar Lavage	Partial improvement
38	Qatar	Mahajan et al. (44)	2021	49/male	No	Gasoline	Fever, cough, chest pain and shortness of breath	NO	Antibiotics And Steroid Therapy	Partial improvement
39	Italy	Arena et al. (45)	2020	65/male	Tracheotomy	Oil-based products	Acute onset of fever, productive cough and dyspnoea	NO	Steroid Therapy	Improved

Summarized in Table 2, we found that ELP has no specific clinical manifestations and is difficult to distinguish from common respiratory diseases. Acute ELP is due to inhalation of large amounts of oily substances in a short period of time. It is often characterized by fever and shortness of breath. Chronic ELP has a long history of exposure to oily substances. Symptoms are dominated by chest pain, dry cough, or even no obvious symptoms, and usually do not manifest as fever or coughing up sputum. Chronic ELP is challenging to diagnose and is easily confused with lung cancer and idiopathic pulmonary fibrosis. We have found that nontuberculous mycobacterial (NTM) infections are susceptible to secondary ELP, mainly *Mycobacterium choriogenes* and *Mycobacterium kansasii* infections (11, 12). Repeated inhalation can lead to pulmonary fibrosis and even hypoxic respiratory failure (13, 14).

There is no uniform treatment protocol for ELP. In addition to immediate stopping of exposure to oily substances, application of steroids has been chosen in most cases. The use of antibiotics has not been found to be more beneficial in the absence of combined bacterial infections. Anti-NTM drugs are used when combined with NTM infection. Surgical resection was chosen for treatment in some cases when the lesion was severe or could not be differentiated from lung cancer. For asymptomatic patients with only imaging manifestations, some cases remain partially improved clinically without treatment. Therefore, in clinical work, we should analyze specific cases and choose appropriate treatment to maximize clinical efficacy.

From Table 2, we found that the prognosis is good for patients without underlying disease. Most clinical symptoms resolve despite slow or even no image absorption. In children, mostly with congenital dysplasia, the disease progresses rapidly and the prognosis is poor. Elderly patients with complex underlying disease or immunodeficiency also have a poorer prognosis. There is currently one case of ELP combined with lung abscess, which underwent open heart surgery and has a fair prognosis. It is worth noting that application of paraffin oil due to constipation was the main cause of the disease. Therefore, risk factors must be strictly evaluated when applying paraffin oil. For people at high risk of aspiration, including dysphagia and gastroesophageal reflux, the use of paraffin oil should be avoided as much as possible.

In conclusion, an 87-year-old male with a history of exposure to paraffin oil was diagnosed with ELP based on radiological and cytological findings. This is the first reported case of ELP due to medical aspiration. Because of the diverse clinical presentation of ELP, its diagnosis requires a combination of risk factor exposure history, radiologic imaging, cytology, and histopathology. For people at high risk of misadministration, a suitable naso-intestinal tube is more appropriate for feeding and medication.

## Data Availability

The original contributions presented in the study are included in the article/supplementary material, further inquiries can be directed to the corresponding author.
